# OCT guided micro-focal ERG system with multiple stimulation wavelengths for characterization of ocular health

**DOI:** 10.1038/s41598-022-07622-5

**Published:** 2022-03-07

**Authors:** Michael Carlson, Sanghoon Kim, Silvia Aparicio-Domingo, Kang V. Li, Ben Puig, Subrata Batabyal, M. Valeria Canto-Soler, Samarendra Mohanty

**Affiliations:** 1 Nanoscope Instruments Inc, 1312 Brown Trail, Bedford, TX 76022 USA; 2 CellSight Ocular Stem Cell and Regeneration Research Program, Department of Ophthalmology, Sue Anschutz-Rodgers Eye Center, University of Colorado School of Medicine, 12800 East 19th Avenue, Aurora, CO 80045 USA; 3 Nanoscope Technologies LLC, 1624 New York Ave, Arlington, TX 76010 USA; 4 Charles C. Gates Center for Regenerative Medicine, University of Colorado School of Medicine, Anschutz Medical Campus, Aurora, CO USA

**Keywords:** Electrophysiology, Retinal diseases

## Abstract

Inherited retinal disorders and dry age-related macular degeneration are characterized by the degeneration and death of different types of photoreceptors at different rate and locations. Advancement of new therapeutic interventions such as optogenetics gene therapy and cell replacement therapies are dependent on electrophysiological measurements at cellular resolution. Here, we report the development of an optical coherence tomography (OCT) guided micro-focal multi-color laser stimulation and electroretinogram (ERG) platform for highly localized monitoring of retina function. Functional evaluation of wild type and transgenic pigs affected by retinal degeneration was carried out using OCT guided micro-focal ERG (μfERG) with selected stimulation wavelengths for S, M and L cones as well as rod photoreceptors. In wild type pigs, μfERG allowed functional recording from rods and each type of cone photoreceptor cells separately. Furthermore, functional deficits in P23H transgenic pigs consistent with their retinal degeneration phenotype were observed, including decrease in the S and M cone function and lack of rod photoreceptor function. OCT guided μfERG based monitoring of physiological function will enable characterization of animal models of retinal degenerative diseases and evaluation of therapeutic interventions at the cellular level.

## Introduction

Inherited retinal disorders including Retinitis Pigmentosa (RP)^1^, Cone-rod dystrophy^2^, Leber Congenital Amaurosis^3^, Choroideremia^4^, Best’s disease and Stargardt disease^5,6^ as well as dry age-related macular degeneration^7^ (dry-AMD) involve dysfunction of the retina and are characterized by the degeneration of different types of photoreceptors. The degree of visual loss increases with ageing^8^ and this is a major concern for our demographic changes towards the elderly population. Dysfunction or death of photoreceptors leads to loss of signals that initiate visual perception. Further, while Retinitis Pigmentosa^9^ is associated with loss of rods preceding loss of cones, the reverse is encountered in cone-rod dystrophy where loss of cones leads to loss of rods. Gene replacement^10^, optogenetic gene therapy^11^, and regenerative cell^12^ (transplant) therapies have promise to restore vision loss in degenerated regions of the retina. Importantly, accurate measurements of visual function would allow for quantitative evaluation of retinal function restoration after therapeutic treatment. This will in turn provide further insight into the efficiency of integration of transduced genes or transplanted cells to enable vision restoration. Full-field flash Electroretinogram (ERG) has enabled measurement of overall retinal function^13^. Yet, this technique may not detect retinal defects at early stages of disease. On the other hand, the large spot size stimulation^14,15^ in the existing focal ERG cannot provide functional information at the resolution necessary to evaluate therapeutic efficacy at the cellular level. Similarly, the multifocal ERG (mfERG) method^16^ that provides a topographical measurement of retinal activity, cannot isolate function at single cell level. Further, the focal ERG and mfERG illumination use white light and therefore, cannot distinguish different cone functions. Hence, there is a clear need for development of ERG based on multi-color micro-focused laser stimulation to discern cellular changes at high resolution, thus allowing critical evaluation of disease progression and therapeutic interventions.

Here, we report functional evaluation of wild type and transgenic pigs affected by retinal degeneration^17^ using optical coherence tomography (OCT) guided micro-focal ERG (μfERG) with selected stimulation wavelengths for S, M and L cones^18^ as well as rod photoreceptors^19^.

## Materials and methods

### Ethics statement

All animal procedures were conducted at the animal facilities at the University of Colorado Anschutz Medical Campus, which is fully accredited by the Association for Assessment and Accreditation of Laboratory Animal Care International (AAALAC). The studies were performed under protocols approved by the University of Colorado-Anschutz Medical Campus Institutional Animal Care and Use Committee (IACUC), and in compliance with the Association for Research in Vision and Ophthalmology (ARVO) statement for the use of animals in ophthalmic and vision research and the ARRIVE guidelines^20^.

### Animal preparation

P23H hybrid transgenic pigs and wildtype littermates (Age > 1 year, N = 4/group) were obtained from the National Swine Resource and Research Center. Pigs were housed in the animal facilities of the University of Colorado Anschutz Medical Campus. Animals were fasted overnight prior to receiving anesthesia for procedures. Pigs were sedated with Ketamine 10–20 mg/kg and Xylazine 2–5 mg/kg IM. The anesthetic drug Propofol was provided intravenously at a dose range of 0.8–1.6 mg/kg as a bolus and/or 3.2–4.4 mg/kg/h as a constant rate infusion to provide multimodal anesthesia. Heart rate, respiration, temperature and blood pressure were continuously monitored while the pigs were under anesthesia. For both groups, 1% Tropicamide/2.5% Phenylephrine was used for pupil dilation, followed by sterilization of the periocular area with 5% povidone-iodine solution and Proparacaine 0.5% for local anesthesia. Sub-Tenon's block with 2% lidocaine (3–5 ml) was administered using a 19-gauge blunt cannula as local anesthesia providing akinesia and analgesia to the eye by blocking the motor nerves and traversing sensory.

### Spectral domain optical coherence tomography guided ERG system: *NS-Neel*

Spectral domain OCT (SD-OCT) is based on spectral domain implementation of the OCT system developed in the OCT research community, and has been in clinical Ophthalmology practice since 1995^21^. NS-Neel is an OCT-guided electrophysiology system from Nanoscope Instruments, Inc which can perform full-field or micro-focal ERG by controlling the stimulation spot size. In the OCT guided stimulation laser-integrated micro-focal ERG device, the OCT beam (λc ~ 860 nm) shares identical optical path with the stimulation laser beam(s) thus providing targeted stimulation while monitoring microstructure of the retina in near real time. For multi-color stimulation of the same spot(s), blue (450 nm), green (520 nm) and red (638 nm) lasers were combined and coupled to a single mode fiber. The OCT system consists of a near infrared (NIR) low coherence source (LCS) which is coupled into a 50:50 Fiber splitter. The visible stimulation laser beam was coupled to the fiber-optic OCT-source arm (second input channel of the fiber splitter) and directed to the sample. The stimulation laser beam emanating from the output of the fiber splitter is collimated by a lens and targeted to selected spots in the retina by micro-electromechanical system (MEMS) scanning mirrors and a pair of telescopic lenses. During identification of retinal morphology and topography, the visible stimulation laser beam is switched off. The beam from the low-coherence source (for OCT), at the output end of the fiber splitter is collimated by the same lens and scanned by the same MEMS-mirrors and telescopic lenses. The reference beam emanating from the other port of the fiber splitter is reflected via the same port by use of a reference mirror. The back-reflected sample beam from the eye (and retina) and the reference beam are routed back via the fiber splitter to a spectrometer, which comprises grating and lenses. The interferogram is recorded in a line-scan camera and processed to obtain structural information of the retina. The focal laser stimulation spots for micro-focal ERG measurements are marked on the enface OCT image displayed on the viewing screen. To mitigate any scattering from optical components, they are anti-reflection coated. Further, non-reflecting surfaces are angled to avoid multiple reflections.

To make our integrated device user-friendly, we developed a graphical user interface (GUI) software which provides a platform for user interaction, allowing the user to control the OCT and ERG-sensors in order to obtain measurements and view results. The software also has a control panel for changing the power, wavelength, and exposure time of the stimulation laser beam. The stimulation laser beam power at the sample plane can be varied from 0.1 to 10,000 cd s/m^2^ for each individual stimulation wavelength. The location for the micro-focal ERG measurement can then be controlled by the user selecting the desired stimulation locations within the field of view of the enface image. Three-dimensional OCT image rendering was integrated into our software platform to simplify user operation and allow near real-time adjustment of imaging location and acquisition. The micro-focal ERG option is initiated when the user selects any specific locations for stimulation on the enface image and prompts the user with a list of stimulation and recording parameters. After selection of specific parameters such as stimulation power, duration, time between stimulations and number of stimulations, the software guides the user through the beginning of the image/signal recording process which is automated after the startup phase. Processing for micro-focal ERG signal from rods and cones obtained with multiple stimulation wavelengths and intensities is carried out via several steps: filtering (low pass, high pass, and Notch), and averaging (Suppl. Fig. 1).

Prior to SD-OCT imaging using *NS-Neel*, pigs were anesthetized, and eyes prepared as described in animal preparation (above). A lid speculum was placed in the eye. A removable traction suture was placed in the conjunctiva for proper orientation of the eye. Artificial tears or saline was used for ocular surface lubrication. OCT scans covering the entire region of interest (ROI), within the visual streak in the superior region of the retina, were performed. ERG recordings were conducted in a dark room under red light illumination (including display monitor) for visualization of the pig, health monitoring and positioning of the electrodes/optical probe during scotopic measurements. For full-field ERG and micro-focal ERG recording, contact signal electrode was placed on the cornea, with the reference needle located lateral to the eye, and the ground needle located above the eye. After electrode placement, animals were dark adapted for ~ 15 min, and an OCT-based enface image was collected. For full-field ERG, white light stimulation was used. For μfERG, the measurement spots were marked based on anatomy, and focal light stimulation was carried out. Following *Nanoscope Instruments*’ μfERG protocol and using disposable jet electrodes and *NS-Neel* stimulation, ERG responses from different retinal locations were recorded. Light flashes were elicited by pre-selected wavelengths (blue: 450 nm; green: 520 nm and red: 638 nm) of the focused laser beam in *NS-Neel* with a stimulus rate of 0.2 Hz and stimulus duration of 1 ms. Signals were amplified through a Built-In Bias Drive Amplifier, Analog-to-digital converters (ADCs) with a built-in programmable gain amplifier (PGA). Acquisitions were performed at 1 kHz sampling rate, and μfERG signals were filtered using a high-pass filter at 1 Hz and a low-pass filter at 300 Hz with 60 Hz notch filter. Micro-focal ERG from a sequence of 5 light flashes were averaged to obtain the final waveform. After completion of the experiment, all three electrodes were removed from the animal and the eyes were kept moist with a hydrating eye ointment.

### Preparation of eyes for sectioning

At study terminus, pigs were euthanized by initial sedation with Ketamine 10–20 mg/kg and Xylazine 2–5 mg/kg IM, followed by Sodium Pentobarbital (IV 1 ml/10 lbs). Absence of heart rate was confirmed before enucleation. Eyes were enucleated with connected optic nerve and an incision around the sclero-corneal limbus was performed. Eyes were fixed for 2 h in 4% paraformaldehyde in 0.1 M phosphate buffer saline (PBS) pH7.4 (Alfa Aesar part# AAJ19943K2). After 3 washes with 0.1 M PBS, a sucrose gradient was performed leaving the eyes overnight in each of the sucrose’s solution (6.25%, 12.5% & 25%). Eyes were then frozen in 25% sucrose using Methylbutane and dry ice and stored at – 80 °C until use. Eyes were thawed at room temperature (RT) in 25% sucrose and the area of interest dissected and embedded in a mix of 25% sucrose/O.C.T. (Tissue-Tek optimal cutting temperature compound, Sakura catalog number 2021) in a histology mold and frozen using Methylbutane and dry ice. Samples were sectioned using a Cryostat, Microm HM550, and low-profile blades.

### Immunostaining

Immunohistochemistry was carried out in 12-μm cryostat sections. Sections were blocked and permeabilized in 10% goat serum/0.25% Triton X-100 for 2 h at room temperature and incubated overnight at 4 °C in a primary antibody mixture. Primary antibodies used: Recoverin (Millipore, AB5585; 1:500) and Rhodopsin (Abcam, ab98887; 1:200). Antibody binding was detected with Alexa Fluor^®^ 488 AffiniPure Fab Fragment Goat Anti-Mouse IgG (H + L) (Jackson ImmunoResearch Laboratories Inc; code: 115-547-003) and Alexa Fluor^®^ 594 AffiniPure Fab Fragment Goat Anti-Rabbit IgG (Jackson ImmunoResearch Laboratories Inc; code: 111-587-008 (1:1000). Sections were counterstained with DAPI. Images were taken with a Nikon Ti-E PFS C2 LUN-A Confocal Microscope.

### Statistics

GraphPad Prism was used to analyze data. The data were plotted as mean ± S.D. Statistically significant difference analyses were carried out by using the nonparametric Mann Whitney U-test, wherein, n_1_, n_2_ indicates total number of observations in Groups 1 and 2 respectively, U implies number of higher values in all pairwise contests, *P* indicates the measure of the probability that an observed difference occurred by random chance, and *P* < value or *P* > value summarizes a confidence interval used to accept or reject the null hypothesis. We test our hypothesis that the independent groups’ measurements differ from one another with *P* < 0.01 and *P* < 1E−5 considered as statistically significant.

## Results

### OCT guided micro-focal ERG system with multiple stimulation wavelengths

**Figure 1:**
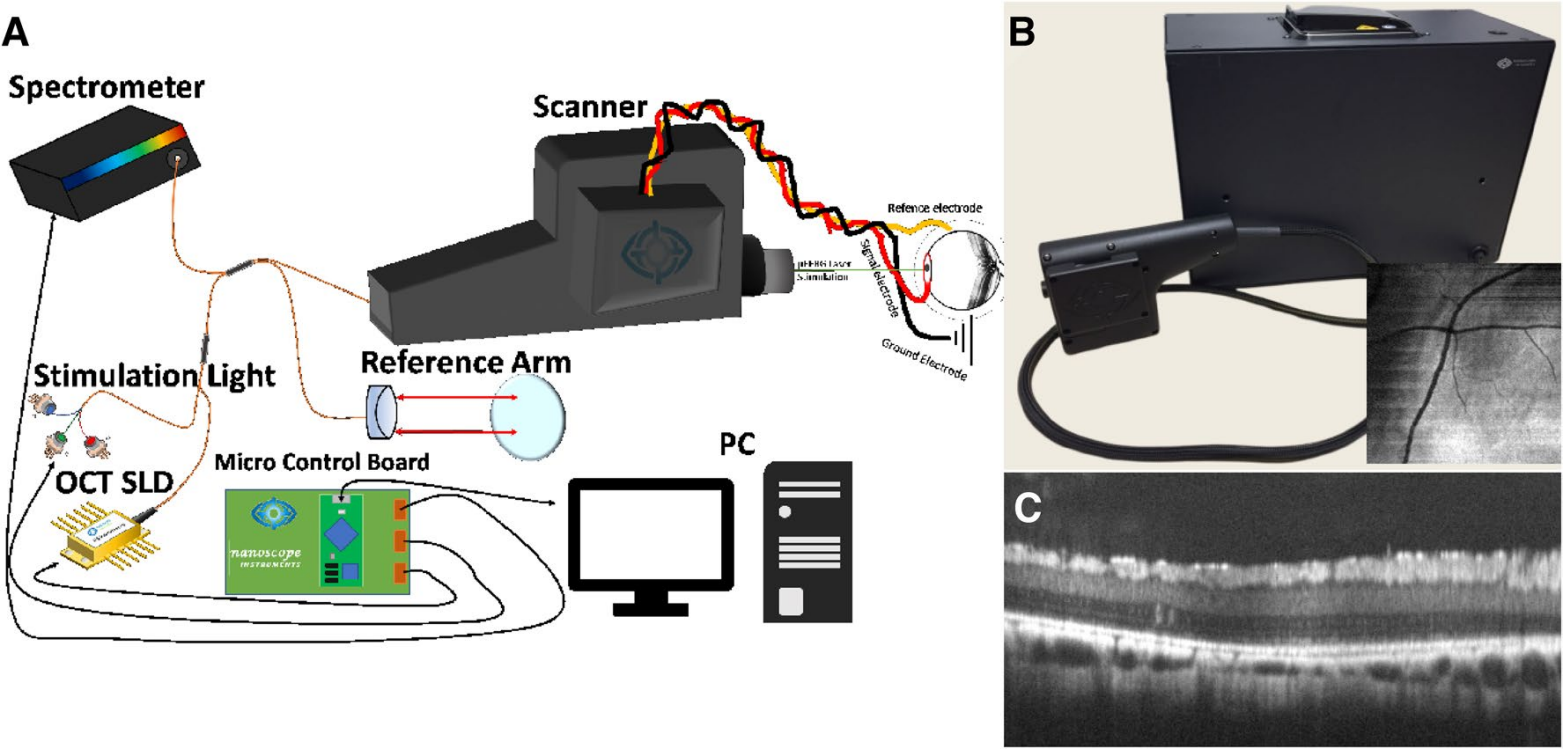
OCT guided micro-focal ERG system with multiple stimulation wavelengths. (**A**) Schematic of the device. (**B**) Picture of the system, Inset: enface image of pig retina generated by C-scan OCT illustrating blood vessels. (**C**) B-scan OCT image of pig retina.

We have developed a first-of-its kind OCT guided micro-focal ERG system capable of multi-color stimulation. The system allows capture of OCT B-scan and enface images which guides selection of areas of interest and positioning of the stimulation spot(s). While using near infrared wavelengths for OCT imaging, the integrated system uses visible wavelengths to stimulate and capture ERG profiles. Figure 1A illustrates the schematic of the device with all its components. The portable system (Fig. 1B) has the versatility to do OCT as well as ERG and includes handheld or mountable options for the scanner head. Location of blood vessels within the enface OCT image (inset in Fig. 1B) enabled quick identification of the location of the field of view (FOV) within the retina. To inspect three-dimensional structure of the retina (for determining presence of photoreceptors) in the acquired FOV, we sliced within the enface image to capture B-scan images (Fig. 1C). The structural information gained from the enface and B-scan images was used to target spots for micro-focal ERG measurements. A graphical user interface (GUI) software providing a user-friendly platform is included with the system. This software allows controlling the OCT and ERG-sensors to obtain measurements and view results, a control panel for changing the power, wavelength, and exposure time of the stimulation laser beam, three-dimensional OCT image for near real-time adjustment of imaging location and acquisition. Detailed description of the hardware and software, parameter setting, and operation of the instrument is presented in methods, “Spectral domain optical coherence tomography guided ERG system: *NS-Neel*” section.

### Immunohistological characterization of wild type and P23H transgenic pig retina

**Figure 2:**
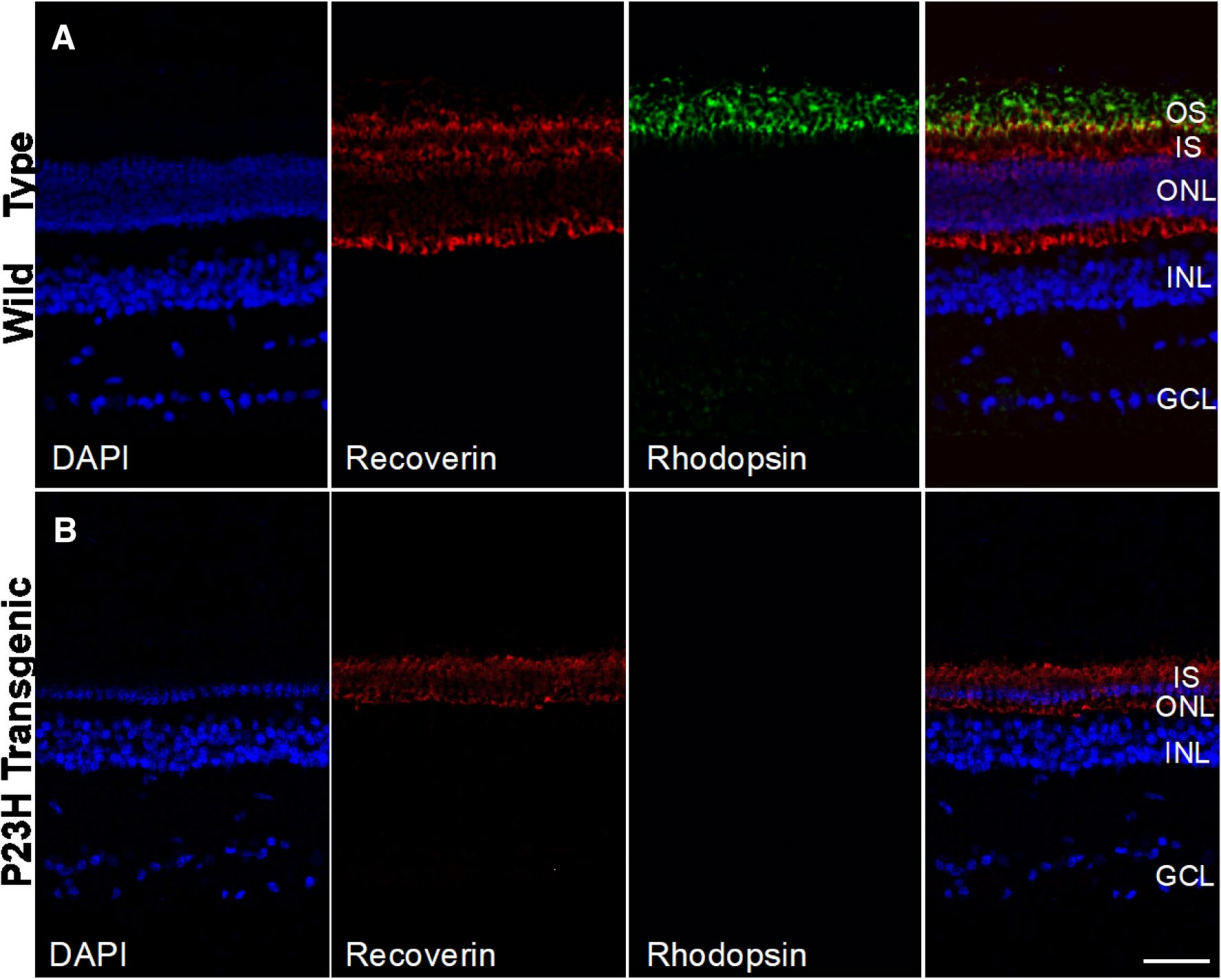
Immunohistochemical labeling of wild type and P23H transgenic pig retina. (**A**) Wild type pig retina displays an outer nuclear layer (ONL) with 8–10 rows of photoreceptor nuclei, including cones (recoverin+/rhodopsin−) and rods (recoverin+/rhodopsin+). (**B**) P23H transgenic retina shows a very thin ONL containing a single row of cones and complete absence of rods; this is accompanied by complete loss of photoreceptor outer segments (OS). *GCL* ganglion cell layer, *INL* inner nuclear layer, *ONL* outer nuclear layer, *IS* photoreceptor inner segments, *OS* photoreceptor outer segments. Scale bar: 50 µm.

Immunohistochemical labeling of wild type and P23H transgenic pig retina was conducted to assess the overall state of the outer nuclear layer, including presence of the different type of photoreceptor cells. P23H transgenic pigs carry the human Pro23His rhodopsin mutation representing the most common form of human autosomal dominant retinitis pigmentosa. As demonstrated in Fig. 2, the retina of wild type pigs contained an outer nuclear layer (ONL) with 8–10 rows of photoreceptor nuclei including both cones and rods (Fig. 2A). In contrast, P23H transgenic pig retinas (Fig. 2B) illustrates a very thin ONL with only a single row of photoreceptor nuclei, accompanied by complete loss of photoreceptor outer segments. Lack of rod photoreceptors in P23H transgenic pigs was evident from the absence of rhodopsin-immunostaining.

### OCT guided ERG measurements with multiple stimulation wavelengths in pigs

**Figure 3:**
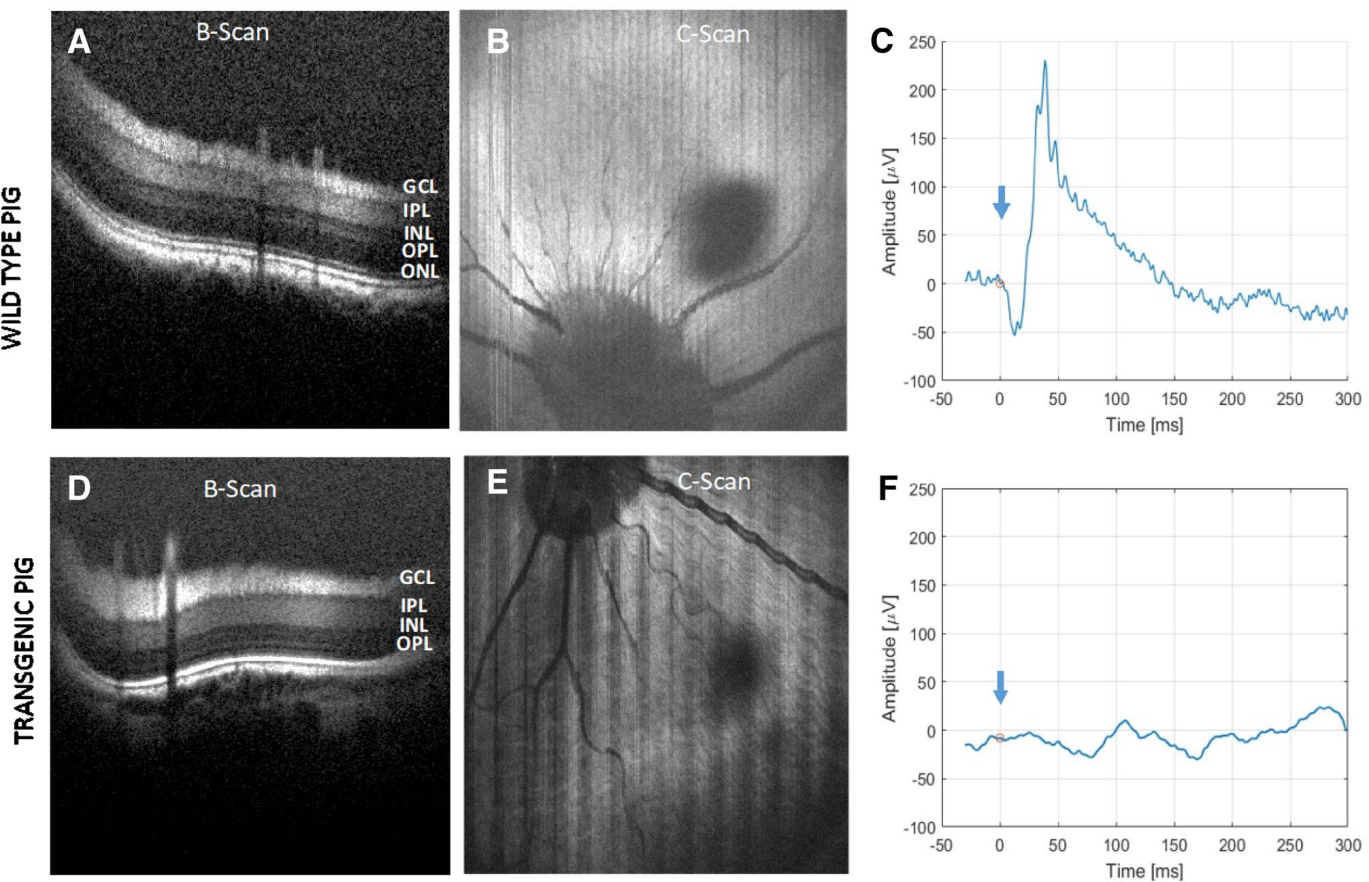
OCT and ERG measurements using an Integrated OCT-Electrophysiology system. (**A**) B-scan OCT image of wild type pig retina illustrates distinct layers. (**B**) Enface image of wild type pig retina reconstructed from C-scan OCT imaging. (**C**) A representative full-field scotopic ERG of wild type pig. (**D**) B-scan OCT image of transgenic P23H pig retina shows loss of ONL. (**E**) Enface image of transgenic P23H pig retina reconstructed from C-scan OCT imaging. (**F**) A representative full-field scotopic ERG of transgenic P23H pig. *GCL* ganglion cell layer, *IPL* inner plexiform layer, *INL* inner nuclear layer, *OPL* outer plexiform layer, *ONL* outer nuclear layer.

Evaluation of retina function in wild type and transgenic pigs was carried out using our integrated NS-Neel OCT-guided electrophysiology system with both, standard full-field ERG and the new modality of microfocal electroretinography (μfERG) with selected stimulation wavelengths for rhodopsin as well as S, M and L cone-opsins. B-scan OCT image of the wild type pig retina displays distinct layers (Fig. 3A). A representative enface OCT image of wild type pig retina is shown in Fig. 3B. Figure 3C illustrates a representative average for full-field scotopic ERG in the wild type pig generated using white light (intensity: 0.1 cd s/m^2^). On the other hand, and in agreement with our immunohistochemical observations (Fig. 2), the B-scan OCT image of transgenic P23H pig retina showed loss of outer nuclear layer (ONL; Fig. 3D). While both wild type and transgenic pigs displayed similar enface images (Fig. 3B,E), they exhibited discernable differences in retinal thicknesses. Measurements of retinal thickness obtained from the B-scan images indicated 271 ± 17 μm for the wild type and 211 ± 11 μm for the transgenic pig, which is in good agreement with previous literature^22^. The measurements were taken at locations in the nasal superior and exhibited significant difference between transgenic and wild type retinal thicknesses (Medians 211 μm and 273 μm, Mann–Whitney U = 31, n_1_ = 35 n_2_ = 55, *P* < 1E−5, *P* = 2.58E−15 two-tailed). No retinal arterial vascular attenuation, optic nerve pallor, or intraretinal pigment migration was observed in the transgenic pig model in the enface image (Fig. 3E). However, the full-field scotopic ERG in transgenic pigs demonstrated no detectable response (Fig. 3F). The lack of transgenic pig ERG response is caused by the discernable ONL shrinking/disappearance observed in the B-scan OCT image and immunohistochemical analysis.

**Figure 4:**
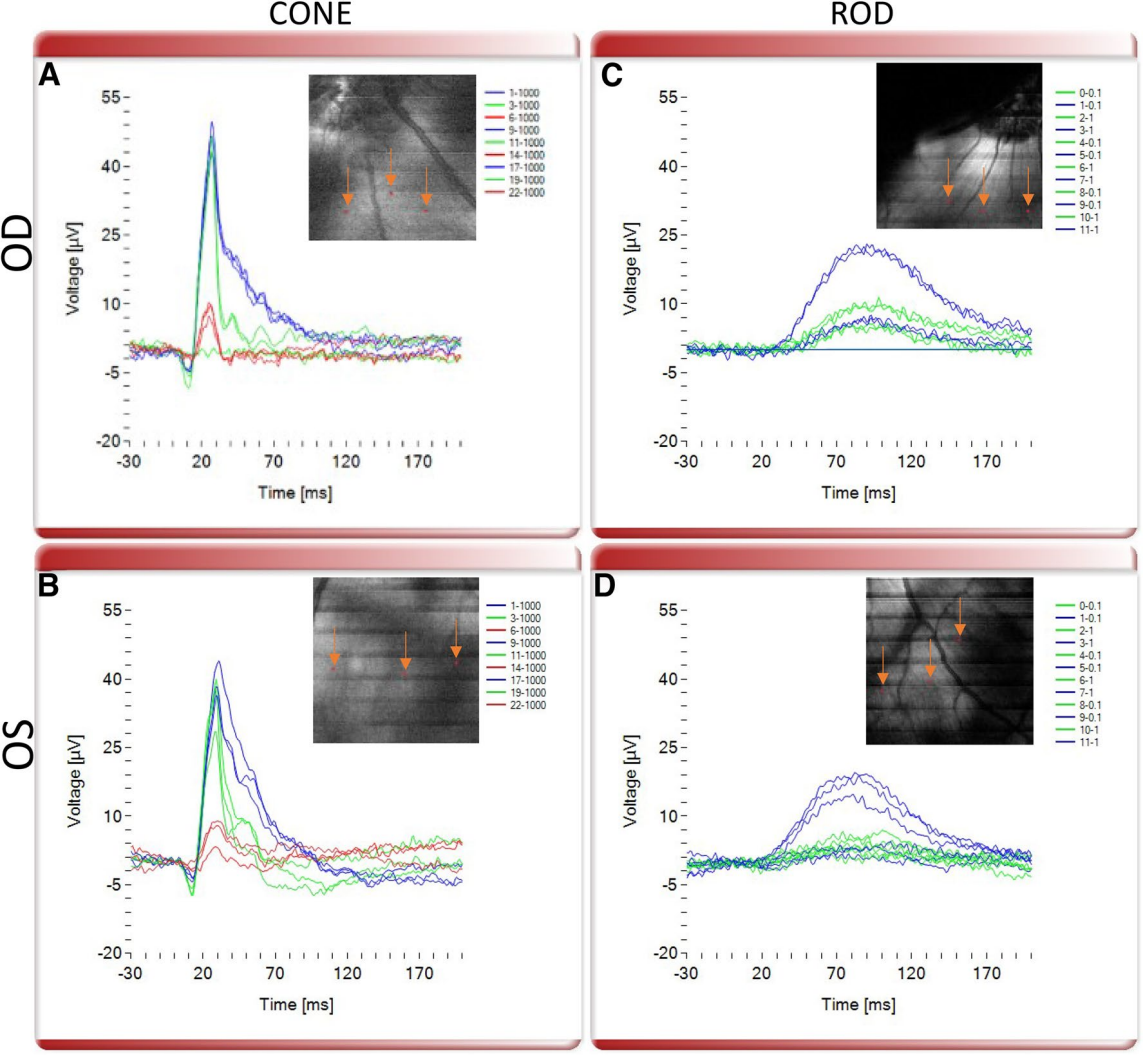
OCT guided micro-focal ERG measurements with multiple stimulation wavelengths in wild type pig. Representative micro-focal ERG of S, M and L cones stimulated by blue (450 nm), green (520 nm) and red (638 nm) focused laser beam with intensity 1000 (cd s/m^2^) at pre-selected points (marked by orange arrows pointed at red dots in the inset OCT enface image) in (**A**) OD and (**B**) OS eyes. Representative micro-focal ERG of rod photoreceptors stimulated by blue (450 nm) and green (520 nm) focused laser beam intensities 0.1 and 1 (cd s/m^2^) at pre-selected points (marked by orange arrows pointed at red dots in the inset OCT enface image) in (**C**) OD and (**D**) OS eyes.

Next, we validated the OCT guided micro-focal ERG system (Fig. 1) for its ability to perform multi-color stimulation of different photoreceptor cell types. OCT images were acquired prior to micro-focal laser stimulation. Figure 4 shows representative results from OCT guided micro-focal ERG measurements with different stimulation wavelengths in wild type pigs. The different cone opsins, S, M and L opsins, were specifically stimulated by blue (450 nm), green (520 nm) and red (638 nm) focused multi-color laser beam at 1000 cd s/m^2^ at pre-selected points as illustrated in Fig. 4A,B. Clear micro-focal ERG responses were observed upon stimulation with the blue and green wavelengths (Fig. 4A,B). As compared to full field ERG, the small spot size of the probed regions led to lower amplitude of the measured signal. Importantly, consistent with the lack of L-cones in the pig retina, minimal micro-focal ERG response was observed with red light stimulation. On the other hand, using lower intensity stimulation than that used to stimulate cones (0.1 and 1 cd s/m^2^), micro-focal ERG measurements from rod photoreceptors could be detected and isolated from cone response since under bright illumination, rod response is saturated unlike cones^23^ (Fig. 4C,D). Consistent with standard ERG recordings, while cone response had a and b-wave features, rod response consisted of only-b-wave. Supplementary Figures 2–4 demonstrate OCT guided micro-focal ERG measurements with multiple stimulation wavelengths in additional wild type pigs.

### Functional deficits in the transgenic pig model evaluated by OCT-guided multi-color μfERG

**Figure 5:**
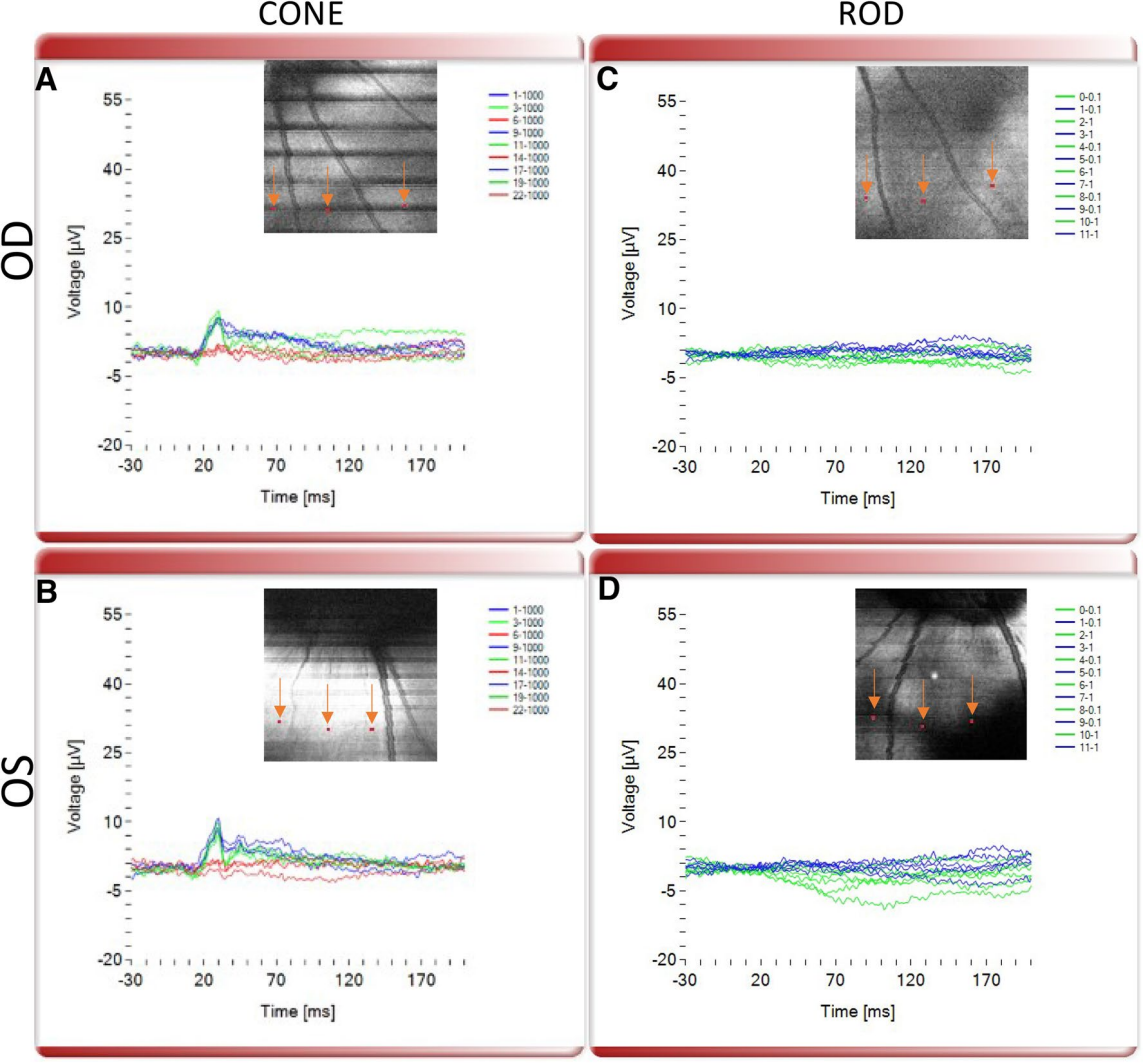
OCT guided micro-focal ERG measurements with multiple stimulation wavelengths in transgenic P23H pig. Representative micro-focal ERG of S, M and L cones stimulated by blue (450 nm), green (520 nm) and red (638 nm) focused laser beam with intensity 1000 (cd s/m^2^) at pre-selected points (marked by orange arrows pointed at red dots in the inset OCT enface image) in (**A**) OD and (**B**) OS eyes. Representative micro-focal ERG of rod photoreceptors stimulated by blue (450 nm) and green (520 nm) focused laser beam intensities 0.1 and 1 (cd s/m^2^) at pre-selected points (marked by orange arrows pointed at red dots in the inset OCT enface image) in (**C**) OD and (**D**) OS eyes.

To further validate performance and sensitivity of our NS-Neel OCT-guided electrophysiology system, we evaluated micro-focal ERG functional responses in the degenerated retina in P23H transgenic pigs. For this, micro-focal ERG recordings subsequent to focal light stimulation at multiple wavelengths were performed. Representative micro-focal ERG responses upon stimulation with multicolor (red, green and blue) focused laser beams at pre-selected points in OD and OS eyes are illustrated in Fig. 5A,B respectively. Compared to micro-focal ERG response in the wild type pigs (Fig. 4), red and green cone responses were severely reduced, consistent with the significantly reduced number of cones present in the transgenic pig retina. Also consistent with the complete absence of rods in the P23H transgenic retina, no rod ERG response was detected (Fig. 5C,D). Supplementary Figures 5–7 show OCT-guided micro-focal ERG measurements with multiple stimulation wavelengths in additional transgenic pigs. In all 4 transgenic pigs, functional deficits consistent with the retinal degeneration phenotype were observed, wherein rod function was absent and, S and M cone function were severely diminished.

**Figure 6:**
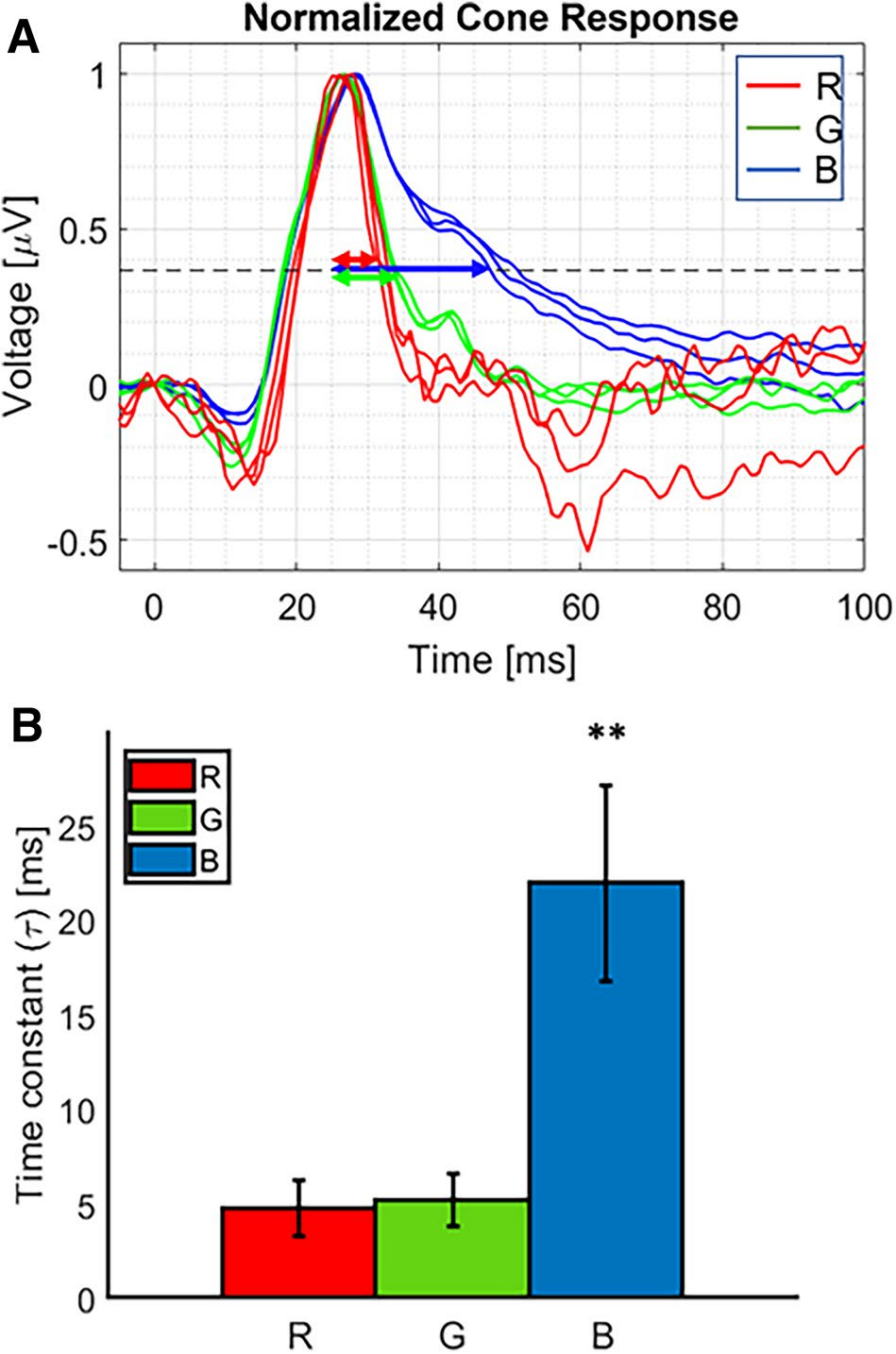
Comparisons of ERG relaxation time (τ) measured with multiple stimulation wavelengths in Wild Type Pigs. (**A**) Representative normalized µfERG of S and M cones stimulated by blue (450 nm), green (520 nm) and red (638 nm) focused laser beam showing respective relaxation time as double-sided arrow. (**B**) Quantitative comparison of relaxation time for different stimulation wavelengths (blue: 450 nm, green: 520 nm, and red: 638 nm). N = 8 eyes. Statistically significant difference between blue vs green or red µfERG relaxation times, **P < 1E−5.

Different stimulation wavelengths spanning the visible light spectrum demonstrated discernable features of the µfERG responses, as illustrated in Fig. 6. The Relaxation times (τ, measured at 1/e, or ~ 0.367 of the normalized height) of cone µfERG for different stimulation wavelengths in wild type pig corresponded to 4.7 ± 1.4 ms, 5.2 ± 1.4 ms, and 22 ± 5.2 ms for blue, green and red stimulation respectively (Fig. 6B). The statistically significant differences between blue vs. green and blue vs. red µfERG relaxation times (Medians 4.7 ms and 5.3 ms, Mann–Whitney U = 0, n_1_ = 22 n_2_ = 21, *P* < 1E−5, *P* = 2.14E−09 two-tailed) and (Medians 4.7 ms and 21.1 ms, Mann–Whitney U = 0, n_1_ = 22 n_2_ = 19, *P* < 1E−5, *P* = 5.01E−08 two-tailed) respectively revealed differential response of S-cone (stimulated by blue light) and M-cone (excited by green and red light). In contrast, the similar µfERG relaxation time measured with green and red stimulation (Medians 5.3 ms and 21.1 ms, Mann–Whitney U = 168, n_1_ = 21 n_2_ = 19, *P* > 0.05, *P* = 0.4011 two-tailed) confirmed excitation of M-cones at these two wavelengths, and lack of L-cone response as expected in the pig retina^24^. On the other hand, there were no statistically significant differences in relaxation times of the rod µfERG response between blue (65.7 ± 20.4 ms) and green (69.1 ± 22.4 ms) stimulation light (Medians 65.5 ms and 70.5 ms, Mann–Whitney U = 118, n_1_ = 19 n_2_ = 14, *P* > 0.05, *P* = 0.5974 two-tailed), owing to the singular nature of the photoreceptor type.

### Quantitative comparison of OCT guided μfERG measurements in wildtype and transgenic pigs

**Figure 7:**
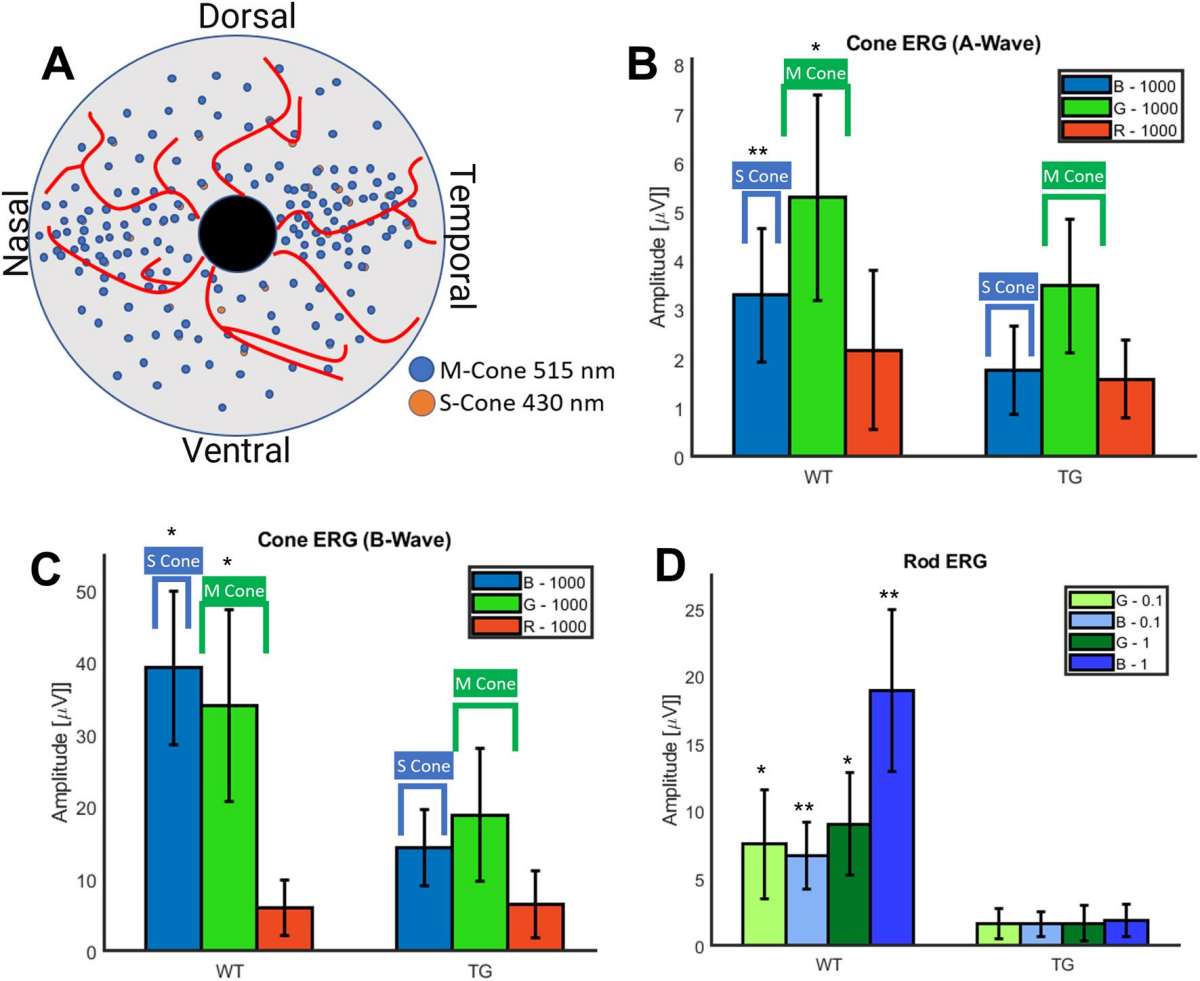
Quantitative comparison of OCT guided micro-focal ERG measurements with multiple stimulation wavelengths in wild type and transgenic pigs. (**A**) Top: schematic of distribution of S and M cones in pig retina. (**B**) Quantitative comparison of A-wave amplitude of micro-focal ERG of S and M-cone photoreceptors stimulated by blue (B, 450 nm) and green (G, 520 nm) focused laser beam in wild type (WT) and transgenic (TG) pigs at light intensity 1000 (cd s/m^2^). (**C**) Quantitative comparison of B-wave amplitude of micro-focal ERG of S and M-cone photoreceptors stimulated by blue and green focused laser beam in WT and TG pigs at light intensity 1000 (cd s/m^2^). Red (638 nm) micro-focal stimulated ERG in WT and TG pigs shows roughly noise level amplitude only at 1000 (cd s/m^2^) indicating absence of L-cones. (**D**) Quantitative comparison of B-wave amplitude of micro-focal ERG of rod photoreceptors stimulated by blue and green focused laser beam in WT and TG pigs at different light intensities 0.1 and 1 (cd s/m^2^). N = 8 eyes/group. Graphs presented as mean ± SD, significance denoted by *P < 0.01 and **P < 1E−5 between wild type (WT) and transgenic (TG) groups.

Schematic of distribution of S and M cones in the pig retina^25^ is illustrated in Fig. 7A. To compare spectral μfERG signals between wild type and degenerated retina, we selected stimulation spots around the area centralis, guided by OCT imaging. Visual illumination with center wavelengths in blue (450 nm), green (520 nm) and red (638 nm) over intensities (0.1 and 1 cd s/m^2^) for rod stimulation or (1000 cd s/m^2^) for cone stimulation to each spot was presented separately for 1 ms, with ≥ 5 s between stimuli. Figure 7 demonstrates quantitative comparison of OCT guided micro-focal ERG measurements with multiple stimulation wavelengths in wild type and transgenic pigs. Figure 7B shows the quantitative comparison of A-wave amplitude of micro-focal ERG of S and M-cone photoreceptors stimulated by blue and green focused laser beam in wild type and transgenic pigs at light intensity (1000 cd s/m^2^). In Fig. 7C, we display quantitative comparison of B-wave amplitude of micro-focal ERG of S and M-cone photoreceptors stimulated by blue and green focused laser beam in wild type and transgenic pigs.

Statistically significant differences in rod and cone functions between wildtype and transgenic pigs were observed. Further, the OCT guided multicolor μfERG measurements allowed discernable variation of electrophysiological response from the pig retina owing to the spatial distribution of photoreceptors (i.e., higher density in visual streaks, as illustrated in Fig. 7A). Statistical analysis using Mann Whitney test showed significant differences between the measured μfERG signal of wild type and transgenic pigs. The statistically significant difference between the cone μfERG a-wave amplitudes of the wild type and transgenic pigs for blue and green stimulation wavelengths corresponded to (Medians 42.2 μV and 13.7 μV, Mann–Whitney U = 7, n_1_ = 19 n_2_ = 22, *P* < 1E−5, *P* = 1.38E−07 two-tailed) and (Medians 38 μV and 15.3 μV, Mann–Whitney U = 72, n_1_ = 19 n_2_ = 22, *P* < 0.01, *P* = 3.59E−04 two-tailed) respectively. Furthermore, differences between the cone μfERG a-wave amplitudes of the wild type and transgenic pigs for red stimulation wavelengths are not statistically significant (Medians 5.36 μV and 5.26 μV, Mann–Whitney U = 221, n_1_ = 19 n_2_ = 22, *P* > 0.05, *P* = 0.76 two-tailed). Similarly, the difference between the cone μfERG b-wave amplitudes of the wild type and transgenic pigs for blue (Medians 3.32 μV and 1.74 μV, Mann–Whitney U = 73, n_1_ = 19 n_2_ = 22, *P* < 0.01, *P* = 3.96E−4 two-tailed) and green (Medians 5.5 μV and 3.2 μV, Mann–Whitney U = 104, n_1_ = 19 n_2_ = 22, *P* < 0.01, *P* = 6.3E−3 two-tailed) stimulation wavelengths were statistically significant unlike the red light (Medians 2.1 μV and 1.6 μV, Mann–Whitney U = 159, n_1_ = 19 n_2_ = 22, *P* > 0.05, *P* = 0.20 two-tailed). Since scotopic response in pigs have a peak flat response from 450 to 520 nm^25^, quantitative comparison of B-wave amplitude of micro-focal ERG from rod photoreceptors stimulated by 450 nm and 520 nm focused laser beam of different light intensities 0.1 and 1 (cd s/m^2^) in wild type and transgenic pigs was also carried out (Fig. 7D). A statistically significant difference was noted for the B-wave amplitude of μfERG for rods of wild type and transgenic pigs with blue light stimulation intensities 0.1 (cd s/m^2^) and 1 (cd s/m^2^) and green light stimulation intensities 0.1 (cd s/m^2^) and 1 (cd s/m^2^), corresponding to (Medians 6.9 μV and 1.4 μV, Mann–Whitney U = 23, n_1_ = 16 n_2_ = 22, *P* < 1E−5, *P* = 6.52E−6 two-tailed), (Medians 16.9 μV and 2 μV, Mann–Whitney U = 0, n_1_ = 16 n_2_ = 22, *P* < 1E−5, *P* = 2.12E−7 two-tailed), (Medians 7.2 μV and 1.3 μV, Mann–Whitney U = 40, n_1_ = 16 n_2_ = 22, *P* < 0.01, *P* = 6.17E−5 two-tailed), and (Medians 9.9 μV and 1.1 μV, Mann–Whitney U = 38, n_1_ = 16 n_2_ = 22, *P* < 0.01, *P* = 4.79E−5 two-tailed) respectively. Red (638 nm) micro-focal stimulated ERG in wild type and transgenic pigs showed no significant difference or detectable amplitude above noise level at light intensity of 1000 cd s/m^2^, consistent with the absence of L-cones.

Supplementary Figure 8 shows quantitative comparison of OCT guided micro-focal multi-color ERG measurements in wild type and transgenic pigs. Quantitative comparison of A-wave amplitude of micro-focal ERG of S and M-cone photoreceptors stimulated by blue (450 nm) and green (520 nm) focused laser beam in OD and OS eyes in wildtype and transgenic pigs at different light intensities 100, 1000, and 10,000 (cd s/m^2^) is demonstrated in Suppl. Fig. 8A. Quantitative comparison of B-wave amplitude of micro-focal ERG of S and M-cone photoreceptors stimulated by blue and green focused laser beam in wild type and transgenic pigs at different light intensities is illustrated in Supplementary Fig. 8B. Red (638 nm) micro-focal stimulated ERG in wild type and transgenic pigs showed detectable amplitude only at highest light intensity (10,000) once again consistent with the absence of L-cones. Supplementary Figure 8C displays quantitative comparison of B-wave amplitude of micro-focal ERG of rod photoreceptors stimulated by blue and green focused laser beam in wild type and transgenic pigs at different light intensities 0.1 and 1 (cd s/m^2^). With control in stimulation laser intensity and light versus dark adaptation, our NS-Neel OCT guided μfERG systems allowed discerning statistically significant differences in rod and cone functions.

## Discussion

We have developed a first-of-its kind OCT guided micro-focal ERG system capable of multi-color stimulation. The NS-Neel OCT guided μfERG is a portable system allowing for structural and functional measurements simultaneously. It includes capabilities for standard B-scan and enface OCT imaging as well as standard ffERG. Notably, the μfERG system also provides focal light stimulation in very discrete areas (approximatively 15 μm) using a variety of wavelengths, thus allowing for measuring electrophysiological response of specific photoreceptor types based on the wavelength in localized areas, determined by OCT imaging. Based on the joint capabilities of the system, structural features or changes can accurately be defined in a localized area via OCT, while functional properties can be correlated with three-dimension structural features in real-time. Importantly, the portability of the system and the characteristics of the wavelengths and intensities used, makes the NS-Neel system suitable for use in point-of-care clinics with a minimal footprint.

Swine, next to nonhuman primates (NHP), are the most human-like neurological models available to date and are increasingly being used as an alternative to NHP for translational preclinical studies^26–28^. Similar to humans, pigs have binocular vision, and the retina is fully developed at birth and adapted to a diurnal activity mainly using cones^27,29^. Importantly, the pig retina has a rod-enriched periphery and a cone-enriched area centralis, also referred as visual streak, that resembles the human macula^29–31^. Over the past decade, the use of the minipig in particular as preclinical model has significantly expanded^27,28^, and existing studies specifically support the suitability of the minipig for assessment of therapeutics targeting ocular disorders^32–37^. The P23H transgenic pig in particular, provided a unique model for testing and validation of the NS-Neel μfERG system. P23H transgenic pigs carry the human Pro23His rhodopsin mutation representing the most common form of human autosomal dominant retinitis pigmentosa. Like the human condition, this mutation leads to loss of rods followed by loss of cones, with a single row of cones remaining within the area centralis at later stages of the disease^26–28^. Our results in both, wild type and P23H pigs, demonstrate that the NS-Neel OCT-guided μfERG with selected stimulation wavelengths allows functional evaluation of different types of cones as well as rod photoreceptors in normal and degenerated retina. The capability of the system to vary the wavelength of the laser stimulation beam allows, for the first time, probing of different color-sensitive cones. Furthermore, functional deficits observed in the P23H degenerated retina are consistent with the corresponding phenotype, with no detectable rod response in contrast to a clear rod response observed in wild type pigs, and dramatically decreased response from S and M cones compared to the wild type. The ability to accurately measure functional differences between normal and diseased retina that the NS-Neel system provides will allow evaluation of disease progression and efficacy of therapeutic interventions at a level of resolution not feasible to date.

OCT guided μfERG based monitoring of physiological function will allow for improved characterization of both, currently available and new models, as well as the development of experimental protocols requiring simultaneous and accurate tracking of rod, S, M, and L cone function in highly localized areas. In addition, this new system provides the capability of monitoring both structural and functional features simultaneously, with a time resolution within seconds or up to weeks, months, or years. Being able to track structural and functional changes in specific regions of interest within the retina expands the possibilities of experimental protocols within a living animal model to levels of accuracy that border in vitro single cell experiments. Importantly, the changeable lens in the scanner head placement allows performing OCT-guided micro-focal ERG measurements in a wide range of animal models (including pigs, rodents, and non-human primates among others) as well as human patients.

Under conventional full field ERG, it is very difficult to differentiate rod response from that of cones due to the higher sensitivity of rods, and rods occupying non independent space within the retina. The ability of the NS-Neel system to perform multicolor stimulation, with a small spot size (~ 15 μm) and controllable intensities allowed to clearly differentiate the responses from rods versus cones, and from each type of cones separately. The differences in stimulation wavelength dependent cone µfERG relaxation time provided discrimination of functional responses from different cone types. Furthermore, at fixed stimulation wavelength (blue or green), the µfERG relaxation time for cones and rods differed, thus allowing differentiation of rod vs cone response. In addition, unlike µfERG, conventional fundus-based mfERG cannot define a or b wave response signals due to the use of rapid stimulation of multiple spots at the same time^38^. Further, mfERG systems utilize multiple-spot stimulations, wherein each stimulation spot has a larger area than used in the μfERG system described here. Therefore, it is not feasible to measure ERG response from highly localized regions of interest using conventional mfERG. Furthermore, fundus mfERG systems are limited in their ability to acquire depth-resolved structural information comparable to that obtained with the OCT guided μfERG described in this study.

## Conclusions

Our results demonstrate that OCT-guided micro-focal ERG with selected stimulation wavelengths is suitable for functional evaluation of each type of cone and rod photoreceptors separately, in normal and diseased retina, and with high spatial precision. With control of stimulation laser intensity, wavelength, and light adaptation level, the system allows discerning differences in rod and cone functional response. The functional deficits observed in transgenic pigs where consistent with the corresponding retinal degenerative phenotype. Thus, OCT guided μfERG based monitoring of physiological function might enable improved characterization of animal models of retinal degenerative diseases and evaluation of therapeutic interventions. Further larger studies are needed for the validity of this conclusion. Additional evaluation of the safety of the NS-Neel system is still needed to advance this technology for future application in humans.

## Supplementary Information

Supplementary Information.
