# Publisher Correction: OCT guided micro-focal ERG system with multiple stimulation wavelengths for characterization of ocular health

**DOI:** 10.1038/s41598-022-11166-z

**Published:** 2022-04-28

**Authors:** Michael Carlson, Sanghoon Kim, Silvia Aparicio-Domingo, Kang V. Li, Ben Puig, Subrata Batabyal, M. Valeria Canto-Soler, Samarendra Mohanty

**Affiliations:** 1 Nanoscope Instruments Inc, 1312 Brown Trail, Bedford, TX 76022 USA; 2 CellSight Ocular Stem Cell and Regeneration Research Program, Department of Ophthalmology, Sue Anschutz-Rodgers Eye Center, University of Colorado School of Medicine, 12800 East 19th Avenue, Aurora, CO 80045 USA; 3 Nanoscope Technologies LLC, 1624 New York Ave, Arlington, TX 76010 USA; 4 Charles C. Gates Center for Regenerative Medicine, University of Colorado School of Medicine, Anschutz Medical Campus, Aurora, CO USA

Correction to: *Scientific reports* 10.1038/s41598-022-07622-5, published online 07 March 2022

The original version of this Article contained an error in Figure 7 where in panel (a) the words Dorsal, Temporal, Ventral, and Nasal were mistyped. The original Figure 7 and accompanying legend appear below.

**Figure 7:**
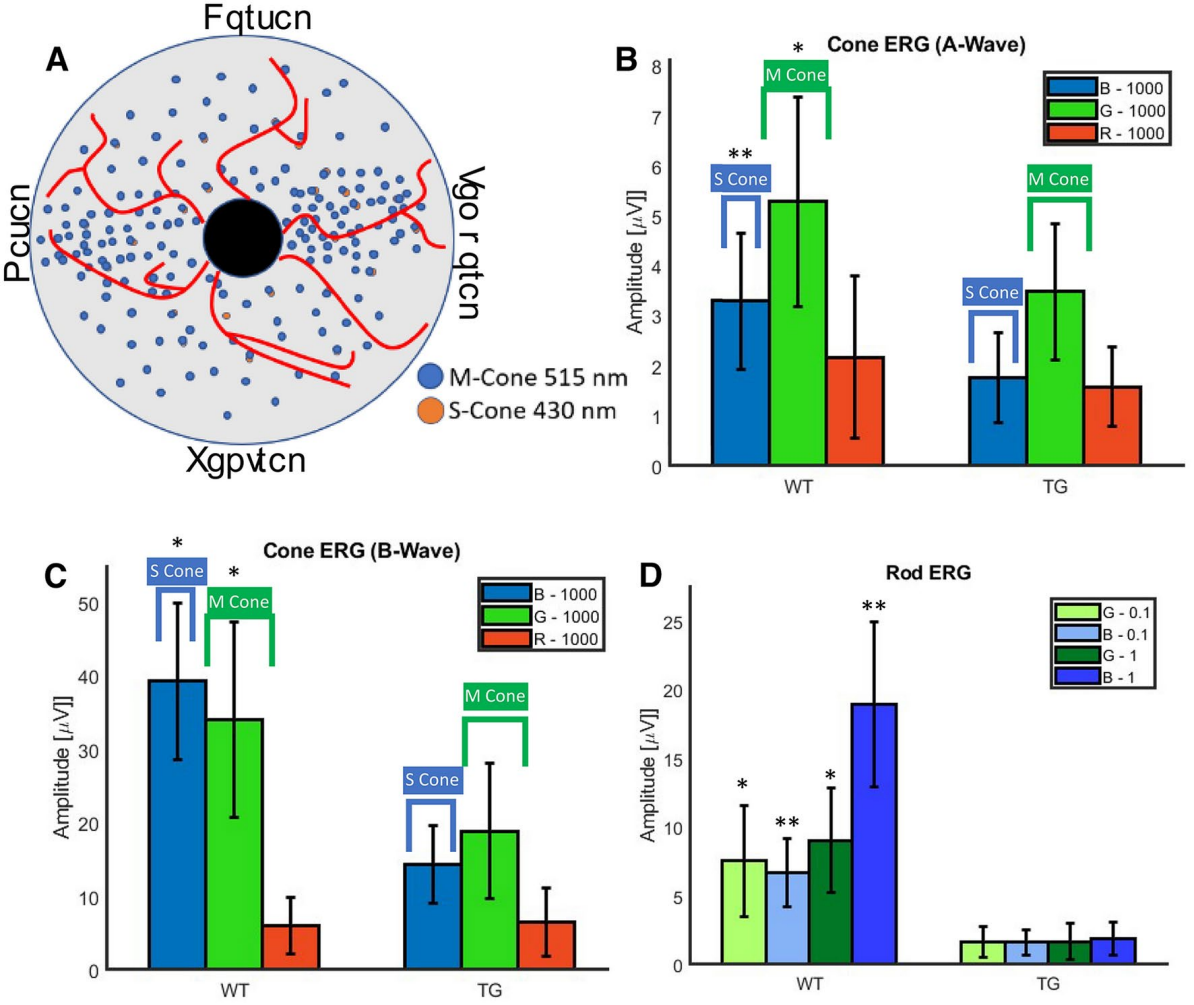
Quantitative comparison of OCT guided micro-focal ERG measurements with multiple stimulation wavelengths in wild type and transgenic pigs. (**A**) Top: schematic of distribution of S and M cones in pig retina. (**B**) Quantitative comparison of A-wave amplitude of micro-focal ERG of S and M-cone photoreceptors stimulated by blue (B, 450 nm) and green (G, 520 nm) focused laser beam in wild type (WT) and transgenic (TG) pigs at light intensity 1000 (cd s/m^2^). (**C**) Quantitative comparison of B-wave amplitude of micro-focal ERG of S and M-cone photoreceptors stimulated by blue and green focused laser beam in WT and TG pigs at light intensity 1000 (cd s/m^2^). Red (638 nm) micro-focal stimulated ERG in WT and TG pigs shows roughly noise level amplitude only at 1000 (cd s/m^2^) indicating absence of L-cones. (**D**) Quantitative comparison of B-wave amplitude of micro-focal ERG of rod photoreceptors stimulated by blue and green focused laser beam in WT and TG pigs at different light intensities 0.1 and 1 (cd s/m^2^). N = 8 eyes/group. Graphs presented as mean ± SD, significance denoted by *P < 0.01 and **P < 1E − 5 between wild type (WT) and transgenic (TG) groups.

The original Article has been corrected.

