# Supplementary material for: OCT guided micro-focal ERG system with multiple stimulation wavelengths for characterization of ocular health
Source: Sci Rep. 2022 Mar 7;12:4009. doi: 10.1038/s41598-022-07622-5 (PMC8901822; doi:10.1038/s41598-022-07622-5)
Supplement: Supplementary file 1 — Supplementary Information. [file 41598_2022_7622_MOESM1_ESM.pdf]

SUPPLEMENTARY INFORMATION

OCT guided micro-focal ERG system with multiple stimulation wavelengths for characterization of ocular health

Michael Carlson[1], Sanghoon Kim[1], Silvia Aparicio Domingo[2], Kang Li[2], Ben Puig[1], Subrata Batabyal[3], M. Valeria Canto-Soler[2,4], Samarendra Mohanty[1,3]*

[1]Nanoscope Instruments Inc, 1312 Brown Trail, Bedford, Texas, USA, 76022

[2] *CellSight* Ocular Stem Cell and Regeneration Program, Department of Ophthalmology, Sue Anschutz-Rodgers Eye Center, University of Colorado School of Medicine, 12800 East 19th Avenue, Aurora, CO 80045

[3]Nanoscope Technologies LLC, 1624 New York Ave, Arlington, Texas, USA, 76010

[4] Charles C. Gates Center for Regenerative Medicine, University of Colorado School of Medicine, Anschutz Medical Campus, Aurora, CO, USA.

Corresponding Author

* E-mail: smohanty@nanoscopetech.com

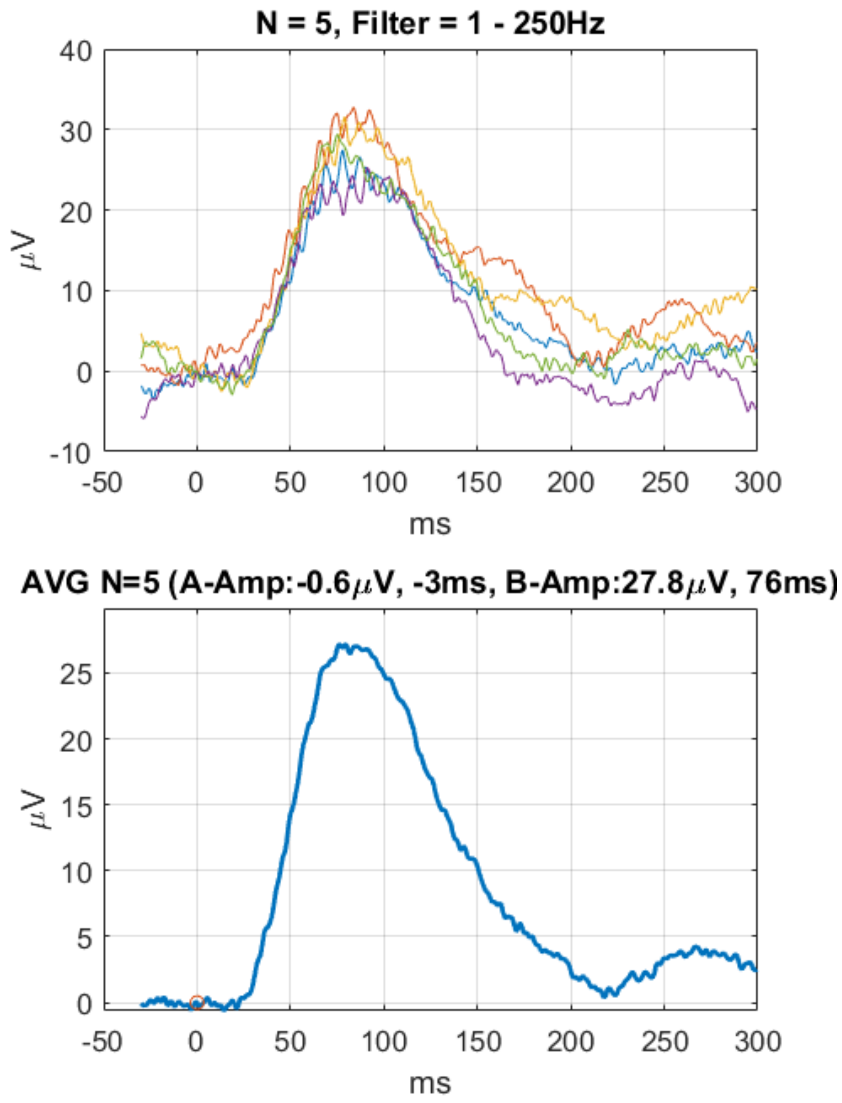

Suppl. Fig. 1. Processing for micro-focal ERG signal from rods and cones obtained with multiple stimulation wavelengths and intensities. Top: Individual micro-focal ERG signal profiles, obtained after 1-250 Hz filtering of raw signals. Bottom: Average of 5 micro-focal ERG signal profiles obtained with repeated stimulations.

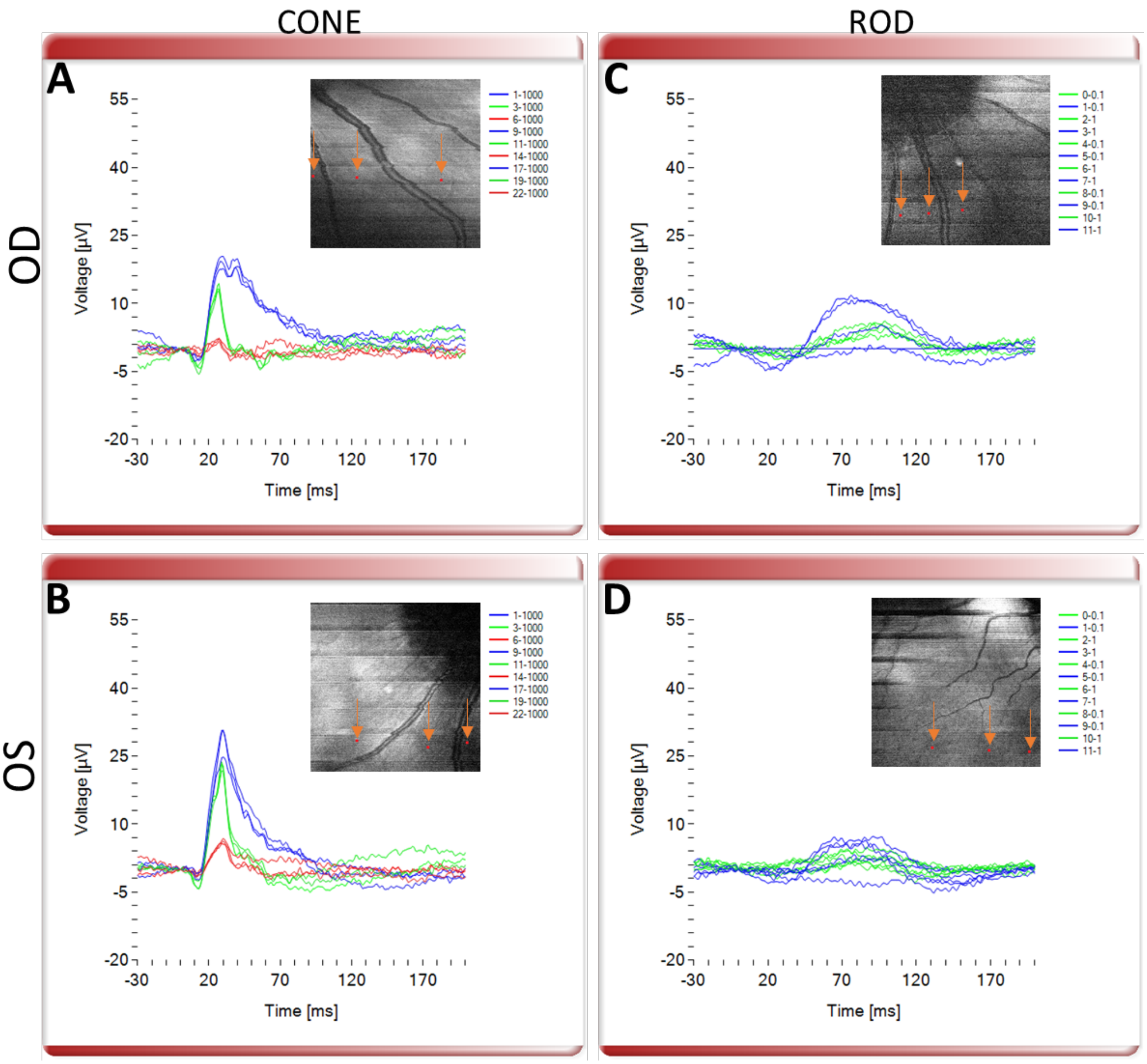

Suppl. Fig. 2. OCT guided micro-focal ERG measurements with multiple stimulation wavelengths in wild type pig-2. Representative micro-focal ERG of S, M and L cones stimulated by blue (450 nm), green (520 nm) and red (638 nm) focused laser beam with intensity 1000 ($cd \cdot s/m^2$) at pre-selected points (marked by orange arrows pointed at red dots in the inset OCT enface image) in (A) OD and (B) OS eyes. Representative micro-focal ERG of rod photoreceptors stimulated by blue (450 nm) and green (520 nm) focused laser beam with intensities 0.1 and 1 ($cd \cdot s/m^2$) at pre-selected points (marked by orange arrows pointed at red dots in the inset OCT enface image) in (C) OD and (D) OS eyes.

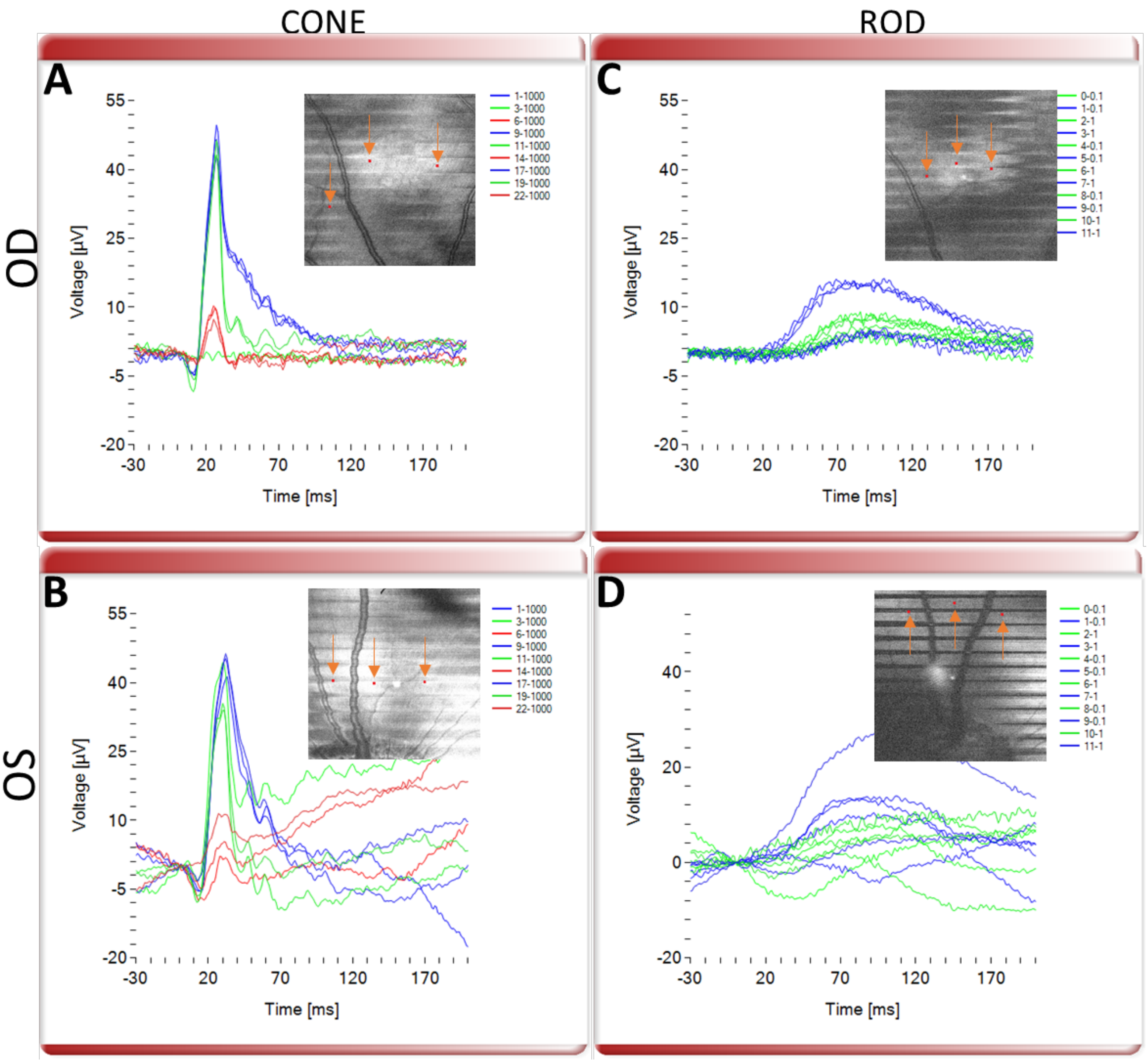

Suppl. Fig. 3. OCT guided micro-focal ERG measurements with multiple stimulation wavelengths in wild type pig-3. Representative micro-focal ERG of S, M and L cones stimulated by blue (450 nm), green (520 nm) and red (638 nm) focused laser beam with intensity 1000 (cd·s/m^2) at pre-selected points (marked by orange arrows pointed at red dots in the inset OCT enface image) in (A) OD and (B) OS eyes. Representative micro-focal ERG of rod photoreceptors stimulated by blue (450 nm) and green (520 nm) focused laser beam intensities 0.1 and 1 (cd·s/m^2) at pre-selected points (marked by orange arrows pointed at red dots in the inset OCT enface image) in (C) OD and (D) OS eyes.

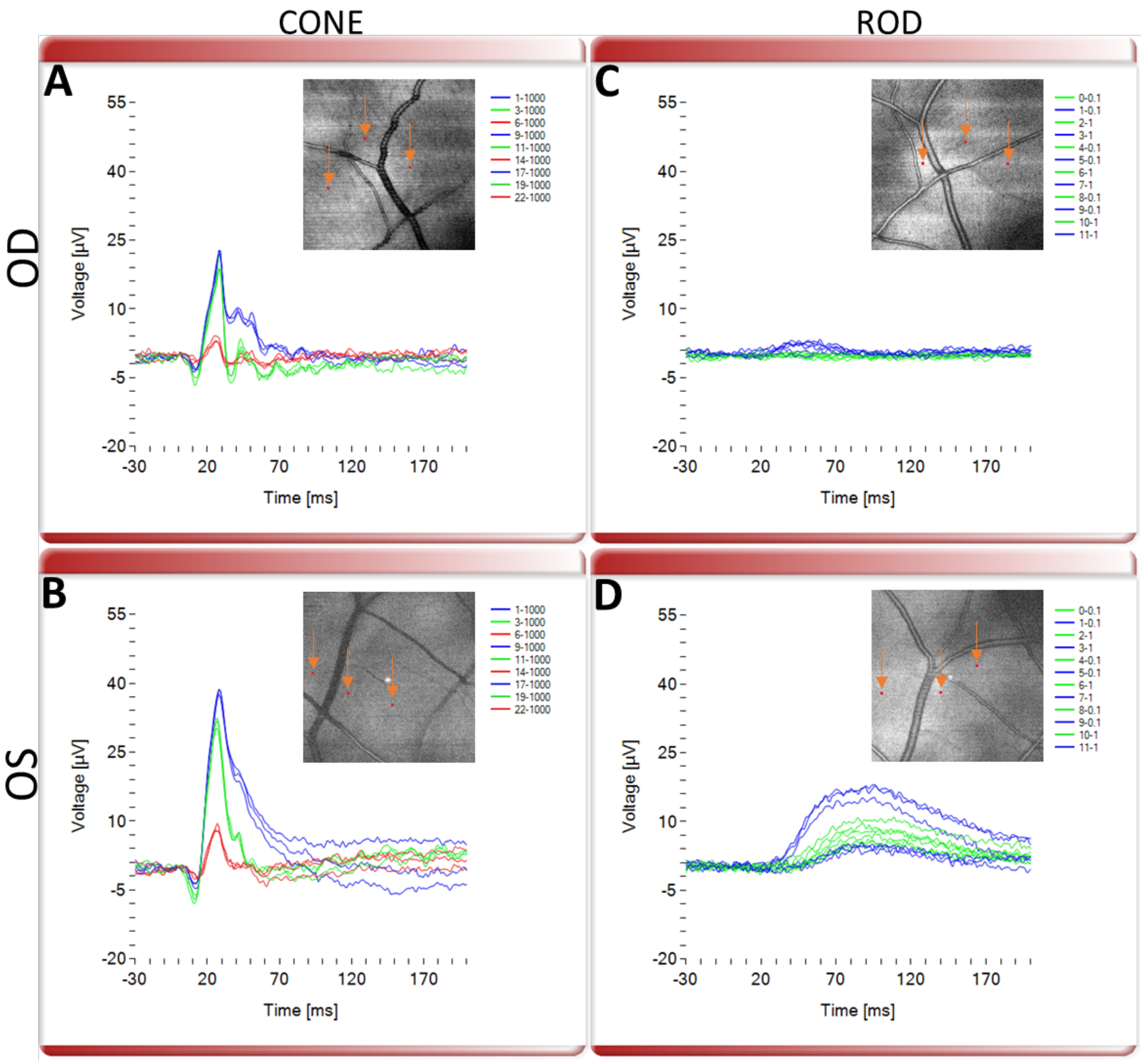

Suppl. Fig. 4. OCT guided micro-focal ERG measurements with multiple stimulation wavelengths in wild type pig-4. Representative micro-focal ERG of S, M and L cones stimulated by blue (450 nm), green (520 nm) and red (638 nm) focused laser beam with intensity 1000 (cd·s/m^2) at pre-selected points (marked by orange arrows pointed at red dots in the inset OCT enface image) in (A) OD and (B) OS eyes. Representative micro-focal ERG of rod photoreceptors stimulated by blue (450 nm) and green (520 nm) focused laser beam intensities 0.1 and 1 (cd·s/m^2) at pre-selected points (marked by orange arrows pointed at red dots in the inset OCT enface image) in (C) OD and (D) OS eyes.

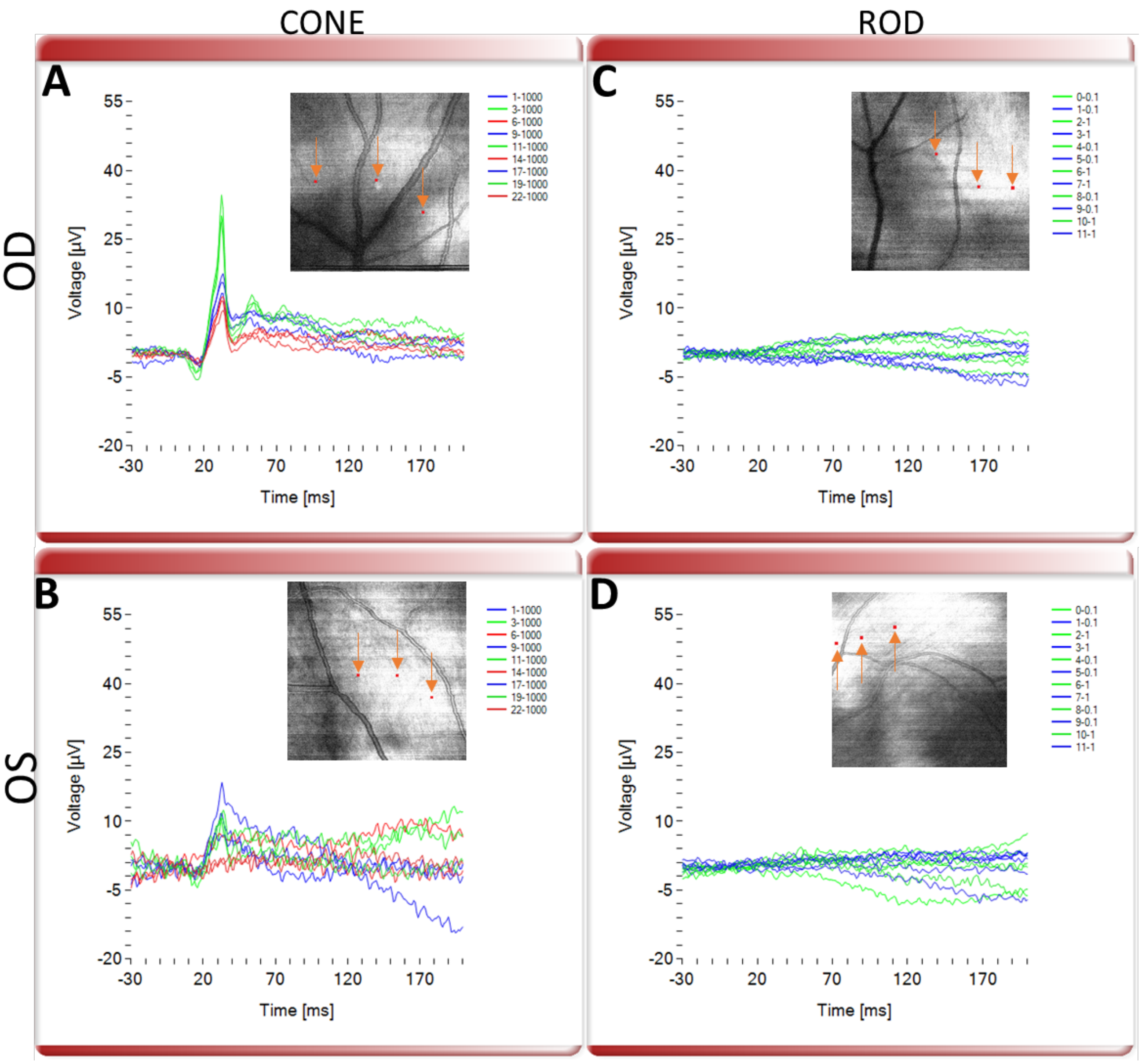

Suppl. Fig. 5. OCT guided micro-focal ERG measurements with multiple stimulation wavelengths in transgenic P23H pig-2. Representative micro-focal ERG of S, M and L cones stimulated by blue (450 nm), green (520 nm) and red (638 nm) focused laser beam with intensity 1000 (cd·s/m^2) at pre-selected points (marked by orange arrows pointed at red dots in the inset OCT enface image) in (A) OD and (B) OS eyes. Representative micro-focal ERG of rod photoreceptors stimulated by blue (450 nm) and green (520 nm) focused laser beam intensities 0.1 and 1 (cd·s/m^2) at pre-selected points (marked by orange arrows pointed at red dots in the inset OCT enface image) in (C) OD and (D) OS eyes.

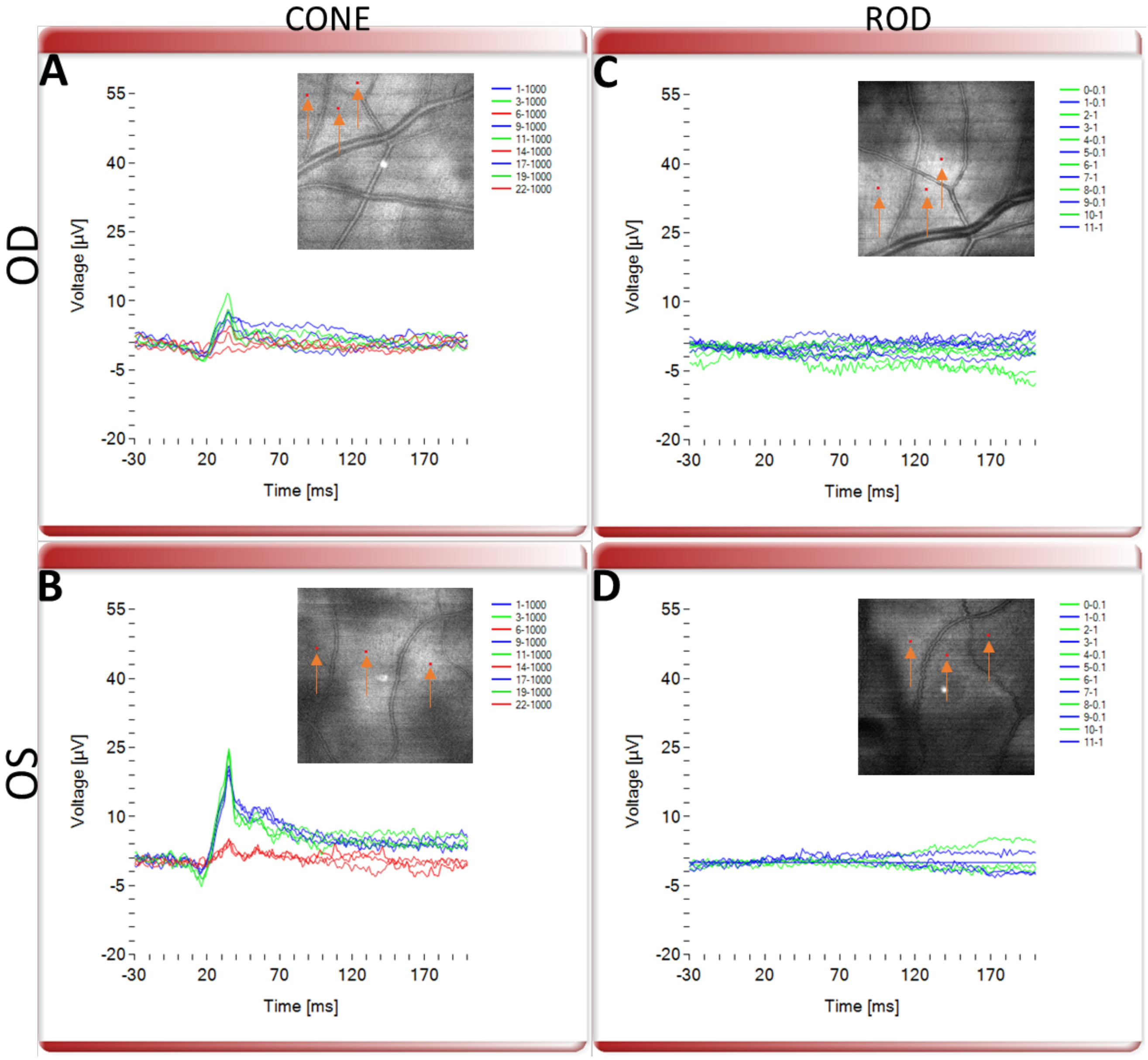

Suppl. Fig. 6. OCT guided micro-focal ERG measurements with multiple stimulation wavelengths in transgenic P23H pig-3. Representative micro-focal ERG of S, M and L cones stimulated by blue (450 nm), green (520 nm) and red (638 nm) focused laser beam with intensity 1000 (cd·s/m^2) at pre-selected points (marked by orange arrows pointed at red dots in the inset OCT enface image) in (A) OD and (B) OS eyes. Representative micro-focal ERG of rod photoreceptors stimulated by blue (450 nm) and green (520 nm) focused laser beam intensities 0.1 and 1 (cd·s/m^2) at pre-selected points (marked by orange arrows pointed at red dots in the inset OCT enface image) in (C) OD and (D) OS eyes.

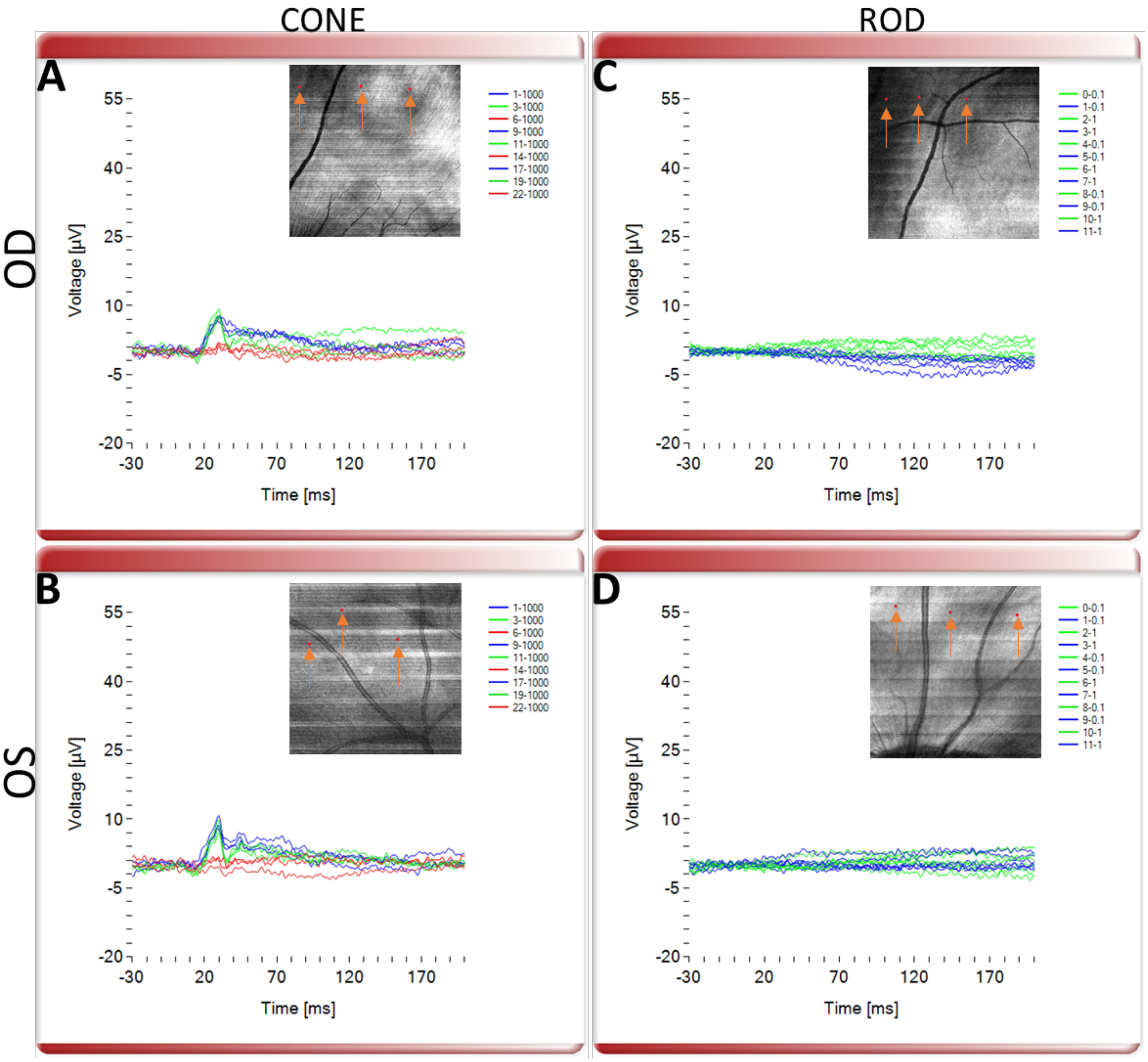

Suppl. Fig. 7. OCT guided micro-focal ERG measurements with multiple stimulation wavelengths in transgenic P23H pig-4. Representative micro-focal ERG of S, M and L cones stimulated by blue (450 nm), green (520 nm) and red (638 nm) focused laser beam with intensity 1000 (cd·s/m^2) at pre-selected points (marked by orange arrows pointed at red dots in the inset OCT enface image) in (A) OD and (B) OS eyes. Representative micro-focal ERG of rod photoreceptors stimulated by blue (450 nm) and green (520 nm) focused laser beam intensities 0.1 and 1 (cd·s/m^2) at pre-selected points (marked by orange arrows pointed at red dots in the inset OCT enface image) in (C) OD and (D) OS eyes.

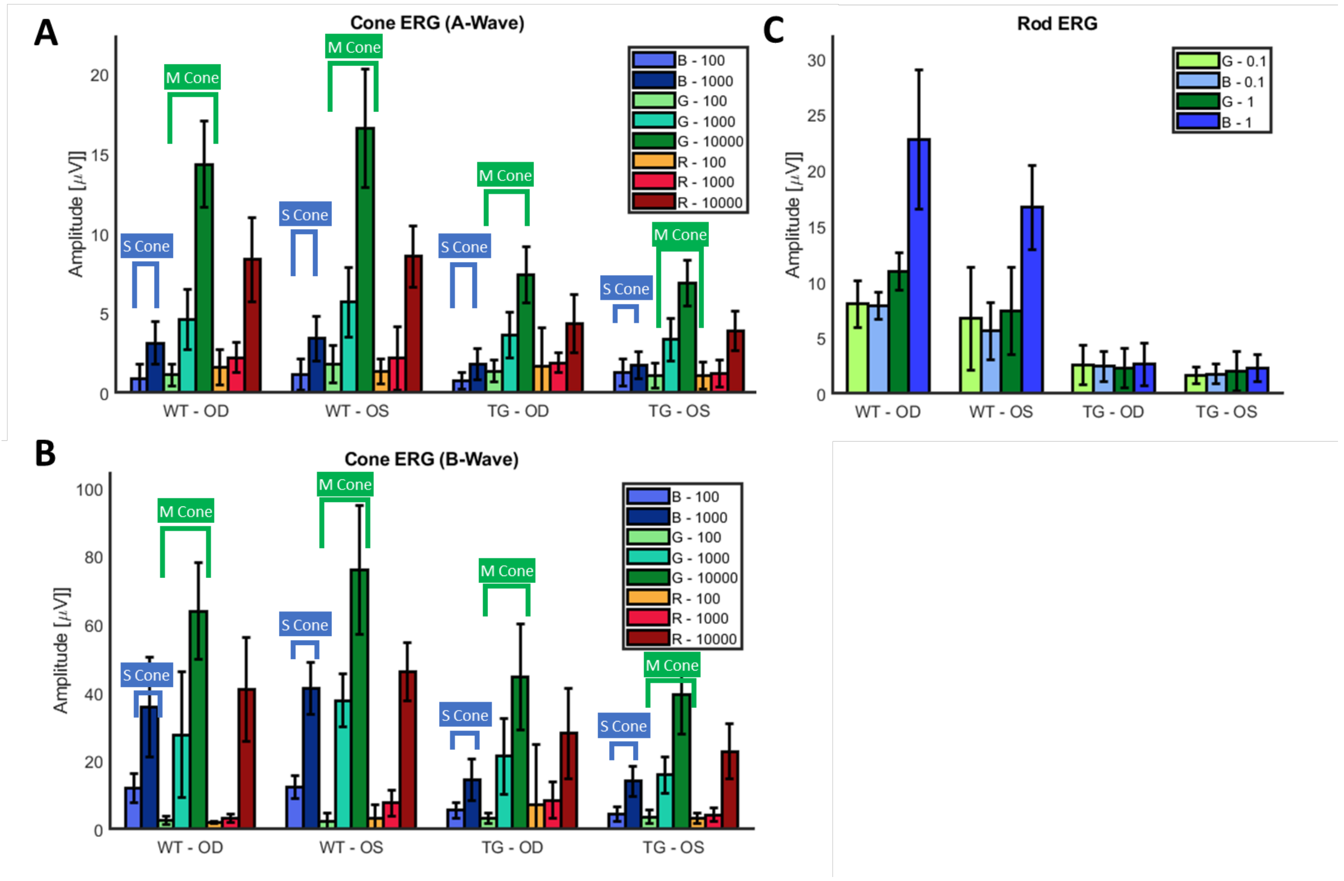

Suppl. Fig. 8. Quantitative comparison of OCT guided micro-focal multi-color ERG measurements in OD and OS eyes of wild type and transgenic P23H pigs. (A) Quantitative comparison of A-wave amplitude of micro-focal ERG of S and M-cone photoreceptors stimulated by blue (B, 450 nm) and green (G, 520 nm) focused laser beam in OD and OS eyes of wild type (WT) and transgenic (TG) pigs at different light intensities 100, 1000, and 10000 ($cd{\cdot}s/m^2$). (B) Quantitative comparison of B-wave amplitude of micro-focal ERG of S and M-cone photoreceptors stimulated by blue and green focused laser beam in OD and OS eyes of WT and TG pigs at different light intensities. Red (638 nm) micro-focal stimulated ERG in WT and TG pigs shows detectable amplitude only at highest light intensity (10,000 $cd{\cdot}s/m^2$) indicating absence of L-cones. (C) Quantitative comparison of amplitude of micro-focal ERG of rod photoreceptors stimulated by blue and green focused laser beam in OD and OS eyes of WT and TG pigs at different light intensities 0.1 and 1 ($cd{\cdot}s/m^2$). N=8 eyes/group, Graphs presented as mean ± SD.
